# A Novel Tandem Mass Spectrometry Method for Rapid Confirmation of Medium- and Very Long-Chain acyl-CoA Dehydrogenase Deficiency in Newborns

**DOI:** 10.1371/journal.pone.0006449

**Published:** 2009-07-30

**Authors:** Frank ter Veld, Martina Mueller, Simone Kramer, Ulrike Haussmann, Diran Herebian, Ertan Mayatepek, Maurice D. Laryea, Sonja Primassin, Ute Spiekerkoetter

**Affiliations:** Department of General Pediatrics, Heinrich- Heine -University, Düsseldorf, Germany; Universidad Peruana Cayetano Heredia, Peru

## Abstract

**Background:**

Newborn screening for medium- and very long-chain acyl-CoA dehydrogenase (MCAD and VLCAD, respectively) deficiency, using acylcarnitine profiling with tandem mass spectrometry, has increased the number of patients with fatty acid oxidation disorders due to the identification of additional milder, and so far silent, phenotypes. However, especially for VLCADD, the acylcarnitine profile can not constitute the sole parameter in order to reliably confirm disease. Therefore, we developed a new liquid chromatography tandem mass spectrometry (LC-MS/MS) method to rapidly determine both MCAD- and/or VLCAD-activity in human lymphocytes in order to confirm diagnosis.

**Methodology:**

LC-MS/MS was used to measure MCAD- or VLCAD-catalyzed production of enoyl-CoA and hydroxyacyl-CoA, in human lymphocytes.

**Principal Findings:**

VLCAD activity in controls was 6.95±0.42 mU/mg (range 1.95 to 11.91 mU/mg). Residual VLCAD activity of 4 patients with confirmed VLCAD-deficiency was between 0.3 and 1.1%. Heterozygous *ACADVL* mutation carriers showed residual VLCAD activities of 23.7 to 54.2%. MCAD activity in controls was 2.38±0.18 mU/mg. In total, 28 patients with suspected MCAD-deficiency were assayed. Nearly all patients with residual MCAD activities below 2.5% were homozygous 985A>G carriers. MCAD-deficient patients with one other than the 985A>G mutation had higher MCAD residual activities, ranging from 5.7 to 13.9%. All patients with the 199T>C mutation had residual activities above 10%.

**Conclusions:**

Our newly developed LC-MS/MS method is able to provide ample sensitivity to correctly and rapidly determine MCAD and VLCAD residual activity in human lymphocytes. Importantly, based on measured MCAD residual activities in correlation with genotype, new insights were obtained on the expected clinical phenotype.

## Introduction

If undiagnosed and untreated, fatty acid oxidation defects (FAOD), such as medium- and very long-chain acyl-CoA dehydrogenase deficiency (MCADD and VLCADD, respectively), are associated with high morbidity and mortality [1]. Clinical presentation of VLCADD may include cardiomyopathy, metabolic encephalopathy, hypoglycaemia and rhabdomyolysis. MCADD is associated with hepatic symptoms that are predominantly related to intercurrent illnesses or prolonged fasting. Thus, early detection by newborn screening (NBS) is absolutely essential for achieving a more favourable outcome and indeed, disease mortality and morbidity is significantly reduced [2], [3].

However, NBS for MCADD and VLCADD using tandem mass spectrometry screening identifies false-positive cases and the outcome of the expanded NBS program in Germany, including MCADD and VLCADD, was recently assessed by Schulze *et al.* demonstrating that the positive predictive values for MCADD and VLCADD were 25.8 and 3.1%, respectively [4]. In contrast to medium-chain acylcarnitines, mild accumulation of long-chain acylcarnitines is observed in healthy children due to activated mitochondrial fatty acid oxidation during catabolism [5]. Furthermore, VLCADD patients may well present with normal confirmatory secondary screening after day 3 of life during anabolism [6]. Taken together, each newborn with an acylcarnitine profile suggestive of MCADD or VLCADD needs further confirmatory diagnosis. Genotyping for prevalent mutations, i.e. the 985A>G mutation in case of MCADD, may represent rapid and cost-effective confirmatory techniques. However, the use of extended, and thus both time-consuming and expensive, mutation screening was reported to be necessary, particularly for suspected MCADD patients who are not of European descent, as recently reviewed by Leonard *et al.* [7]. Likewise, great molecular heterogeneity in VLCADD [8] makes extended mutation screening obligatory in most cases. Genotyping does, therefore, not always constitute a swift and cost-effective confirmatory diagnostic technique for a suspected FAOD in all cases.

In this paper we present a novel, cost-effective and rapid method for the measurement of MCAD and VLCAD activity in human lymphocytes to correctly identify patients and to confirm disease. In addition, we correlate residual MCAD activities with genotype in MCADD.

## Methods

### Participants

Whole blood specimens were obtained from subjects with biochemical indication of MCADD or VLCADD, as identified in NBS, their parents and siblings. As the determination of MCAD and/or VLCAD residual activity constitutes a routine procedure performed by our laboratory, not being part of a scientific study in any way or form, informed consent from subjects, or their parents, was obtained by the referring physicians in oral fashion. The study was performed according to the rules of the Declaration of Helsinki and approved under study number 3230 by the ethical review board of Heinrich-Heine-University Düsseldorf.

### Reagents

Palmitoleoyl-CoA (C16:1-CoA), palmitoyl-CoA (C16:0-CoA) and octanoyl-CoA (C8:0-CoA) were purchased from Sigma (Deisenhofen, Germany) as Li^+^ salts and stored at −20°C. Ferrocenium hexafluorophosphate was obtained from Sigma-Aldrich (Deisenhofen, Germany).

### Procedures

#### Isolation of lymphocytes from blood samples

Lymphocytes were isolated from 2 mL of whole blood using Ficoll-Paque™ Plus (Amersham) and Leucosep ® (Greiner Bio One) tubes as described previously [9].

#### VLCAD and MCAD enzyme assay conditions

Lymphocytes were resuspended in medium (200 µM ferrocenium hexafluorophosphate in 100 mM Tris-HCl or 10 mM NH_4_Ac for HPLC and LC-MS/MS analysis, respectively) to a final protein concentration of 30 µg · mL^−1^. Duplicate enzyme reactions for 5 min. at 37°C and pH = 8.0 were started by adding 4 µL of 5 mM C16:0-CoA as substrate to a total reaction volume of 100 µL. For HPLC analysis, quenching was achieved by adding 10 µL of 2 M HCl, followed by 5 min. of incubation on ice and neutralization with 2 M KOH. For LC-MS/MS analysis, the reaction was quenched by adding 100 µL of −20°C acetonitrile. Subsequently, proteins were removed by precipitation at 13000 rpm for 5 min. and by filtering the supernatant through 96-well 1.2 µm filter plates (Millipore, Schwalbach, Germany). C16:0-CoA was replaced by C8:0-CoA in the MCAD assay, at a concentration of 5 mM.

#### High-pressure-liquid-chromatography analysis

VLCAD assay products, C16:1-CoA and C16:OH-CoA, and the C16:0-CoA substrate were measured based on a method published by Woldegiorgis et al. [10]. Briefly, a Phenomenex C18(2) Luna column (250 mm×4.6 mm×5 µm) in combination with a C18 Luna guard column were used. The two mobile-phase solvents were acetonitrile and 25 mM KH_2_PO_4_, pH = 6.9. Following 2 min. of 70% KH_2_PO_4_ and 30 5 ACN, a three-step linear gradient was used; 1) 70% KH_2_PO_4_ reduced to 60% and 30% acetonitrile increased to 40% over 5 min. 2) 60% KH_2_PO_4_ reduced to 54% and 40% acetonitrile increased to 46% over 9 min. 3) 54% KH_2_PO_4_ reduced to 38% and 46% acetonitrile increased to 62% over 5 min. Next, the 38% KH_2_PO_4_/62% acetronitrile mixture was run for 5 min. Finally, the system was equilibrated for 5 min. The injection volume was 10 µL at a flow rate of 1.5 mL/min. The acyl-CoA esters were detected at 254 nm and 16:1-CoA, C16:OH-CoA and C16:0-CoA eluted at 11,6; 16,6 and 17,5 min, respectively. Quantitation was based on peak areas, with the initial substrate C16:0-CoA amount set to 20 nmol.

#### Tandem mass spectrometry analysis

In quenched VLCAD and MCAD assay samples, C16:1-CoA; C16:OH-CoA; C16:0-CoA and C8:1-CoA; C8:OH-CoA; C8:0-CoA, respectively, were quantified by LC-MS/MS. A Waters 2795 Alliance HPLC system (Waters, Milford, UK), equipped with a thermostated autosampler, was used for solvent delivery and sample introduction. Assay samples were placed in a cooled sample tray (15°C) and 5 µL was injected onto a C18(2) Phenomenex Luna column (100×2.0 mm×3 µm). C16:OH-CoA, C16:1-CoA and C16:0-CoA were eluted isocratically with 55% (v/v) acetonitrile in 10 mM NH_4_Ac (pH was not adjusted and was approx. 6.7) at a flow rate of 200 µL/min. Similarly, C8:OH-CoA, C8:1-CoA and C8:0-CoA were eluted isocratically with 30% (v/v) acetonitrile in 10 mM NH_4_Ac. The eluate was delivered into a Quattro Micro MS/MS (Micromass, Cambridge, UK) with an ESI probe in positive ion mode. Injection interval was 5 min. Nitrogen was used as drying gas at 650 L/h. Collision energy was 35 eV and argon was used as collision gas. Declustering potential was 45 V and ion source temperature was 100°C. Compounds were detected in the multiple reaction monitoring (MRM) mode with the following mass transitions for the VLCAD assay: C16:OH-CoA, m/z 1022.4 → 515.2, C16:1-CoA, m/z 1004.4 → 497.2 and C16:0-CoA, m/z 1006.4 → 499.2, respectively, and C8:OH-CoA, m/z 910.4 → 403.2, C8:1-CoA, m/z 892.4 → 385.3 and C8:0-CoA, m/z 894.4 → 387.2, respectively, for the MCAD assay.

Quantitation was based on peak area ratios, with the initial substrate C16:0-CoA or C8:0-CoA amount set to 20 nmol.

#### Identification of mutations in the ACADM and ACADVL gene

Genomic DNA from whole blood was isolated using DNA Mini kit (Qiagen, Hilden, Germany). Polymerase chain reaction (PCR) was used to amplify all twelve *ACADM* exons, including part of the flanking intron sequences as previously described [11]. *ACADVL*, containing 20 exons, was analyzed according to Spiekerkoetter *et al.* [9]. The amplified DNA fragments were separated by electrophoresis and extracted (QIAquick Gel Extraction kit, Quiagen). PCR products were subjected to DNA sequence analysis using the Cycle sequencing kit, (Applied Biosystems, Weiterstadt, Germany). Analysis was performed using an Applied Biosystems Prism 310 Genetic Analyser.

### Statistical methods

Data were acquired and analysed using MassLynx NT v4.0 (Micromass, UK). Reported data are presented as means±standard error of the mean (s.e.m.). Statistical analyses were performed using Student's t-test. Differences between means were considered significant if *p*<0.05.

## Results

### Characterization and validation of the acyl-CoA dehydrogenase assays

**Figure 1:**
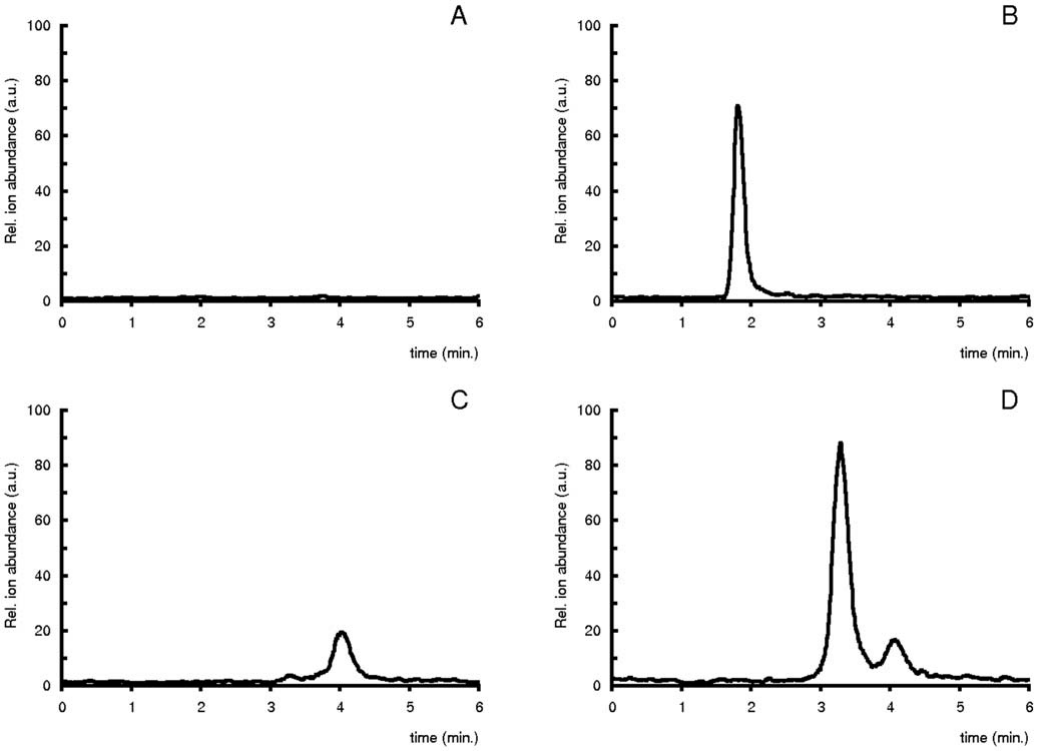
Multiple Reactant Monitoring (MRM) chromatograms of quenched VLCAD assay samples. C16:OH-CoA, m/z 1022.4 → 515.2 (A and B) and C16:1-CoA, m/z 1004.4 → 497.2 (C and D) after 0 (A and C) and 5 (B and D) min. of incubation.

Representative VLCAD assay multiple reactant monitoring chromatograms are shown in Figure 1. The sample was either quenched instantly by adding −20°C acetonitrile, subsequent to substrate (C16:0-CoA) addition (Fig. 1, A and B), or was incubated for 5 minutes at 37°C (Fig. 1, C and D). The hydroxyl-CoA product eluted first (C16:OH-CoA fragment ion, m/z 1022.4 → 515.2) at 1.9 min. and the enoyl-CoA peak (C16:1-CoA fragment ion, m/z 1004.4 → 497.2) eluted at 3.2 min. The observed C16:1-CoA peak eluting at 4.0 min. was independent of assay incubation time or sample amount and was solely dependent on palmitoyl-CoA substrate concentration, most likely identifying it as C16:1-*cis*-9-CoA impurity in the commercial palmitoyl-CoA preparation. As enoyl-CoA products are rapidly converted into hydroxyacyl-CoA by matrix 2-enoyl-hydratases, in order to avoid product inhibition *in-vivo* [12], [13], it was critical to include hydroxyacyl-CoA as acyl-CoA dehydrogenase product to avoid underestimation of catalytic turnover.

The oxidation products in the MCAD assay, C8:OH-CoA and C8:1-CoA, and the added substrate C8:0-CoA had fragment masses of m/z 910.4 → 403.2, 892.4 → 385.3 and 894.4 → 387.2, respectively. MCAD and VLCAD activities in lymphocytes were linear up to 20 min, proportional to the amount of protein added in the range of 10 to 50 µg and were independent of ferrocenium hexafluorophosphate electron acceptor concentrations above 50 µM.

The inter-assay variation (CV) for the HPLC and LC-MS/MS method were determined by measuring the VLCAD activity in the same control lymphocytes on 8 different days and were 9% and 13%, respectively. To determine the intra-assay variation (CV), VLCAD activity was measured in one control lymphocyte sample in triplicate on two consecutive days. Intra-assay variations were 3% and 4% for the HPLC and LC-MS/MS method, respectively.

**Figure 2:**
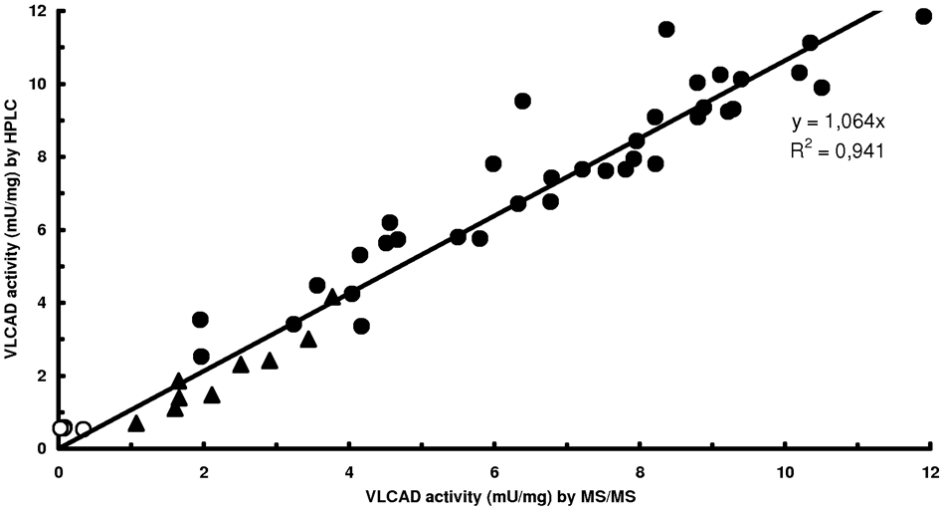
Comparison of results of the standard HPLC assay and the LC-MS/MS assay from the same lymphocyte sample. Total number of subjects tested was 52. (•) Normal controls. (▴) Confirmed heterozygous *ACADVL* mutation carriers. (○) Confirmed homozygous *ACADVL* mutation carriers.

We compared results obtained with our newly developed LC-MS/MS VLCAD assay with VLCAD activities determined by HPLC. In a direct comparison of VLCAD activities in human lymphocytes, the LC-MS/MS method correlates well with the HPLC assay (Fig. 2). We observed that correlation between the established HPLC and newly developed LC-MS/MS method was excellent (r = 0.941) and S_yx_ was±0.804 (Fig. 2).

### Determination of residual VLCAD activities in patients

In total, 52 subjects were measured with our LC-MS/MS VLCAD assay. Four were identified as VLCADD patients and 9 were heterozygous carriers of an *ACADVL* mutation (Fig. 2). VLCAD activities measured by LC-MS/MS in the samples collected from normal subjects (n = 36) ranged from 1.95 to 11.91 nmol · min^−1^ · mg protein^−1^, with a mean±SE value of 6.95±0.42 nmol · min^−1^ · mg protein^−1^ (Fig. 2). Average residual VLCAD activities of four confirmed patients, being homozygous carriers of an *ACADVL* mutation, was 0.13±0.07 nmol · min^−1^ · mg protein^−1^, representing residual activities between 0.02 and 0.34 nmol · min^−1^ · mg protein^−1^ (Fig. 2). In confirmed heterozygous carriers of an *ACADVL* mutation, residual VLCAD activity ranged from 1.07 to 3.77 nmol · min^−1^ · mg protein^−1^. VLCAD activity measurements with HPLC revealed comparable results, but homozygous *ACADVL* mutation carriers had a higher average residual VLCAD activity of 0.55±0.01 nmol · min^−1^ · mg protein^−1^ (Fig. 2).

### Determination of residual MCAD activities in patients

MCAD activities measured by LC-MS/MS in the samples collected from normal subjects (n = 6) ranged from 1.82 to 2.78 nmol · min^−1^ · mg protein^−1^, with a mean±SE value of 2.38±0.18 nmol · min^−1^ · mg protein^−1^. Table 1 lists 28 examined cases that were assayed for MCAD activity, parents of newborns are highlighted. With the exception of cases 4, 6 and 8 all patients with residual MCAD activities below 2.5% were homozygous carriers of the 985A>G mutation. MCADD patients with other mutations had higher MCAD residual activities, ranging from 5.7 to 13.9%. Patients carrying one 199T>C mutation presented with residual activities above 10%. Confirmed 985A>G heterozygotes (cases 20, 22 and 23) had residual MCAD activities above 24% of controls (Table 1).

**Table 1:** Octanoyl-CoA oxidation, plasma medium-chain acylcarnitine levels and gene analysis of both *ACADM* alleles in subjects with suspected MCAD-deficiency.

Case	MCAD (%)^1^	Allel 1	Allel 2	C8:0 I (µM)^2^	C8:0 II (µM)^2^
1	0.4%	985A>G	985A>G	21.00	6.30
2	0.5%	985A>G	985A>G	12.50	3.13
3	0.5%	985A>G	985A>G	19.70	2.64
4	0.6%	245ins>T	IVS9+2T>C^5^	6.00	
5	1.0%	985A>G	985A>G	0.97	0.85
6	1.1%	985A>G	347G>A	3.75	4.76
7	1.1%	985A>G	985A>G		
8	1.3%	245ins>T	IVS9+2T>C^5^	7.73	
9	1.7%	985A>G	985A>G	8.70	2.80
10	1.7%	985A>G	985A>G	12.00	1.42
11	2.3%	985A>G	985A>G	3.40	2.30
12	5.7%	985A>G	IVS^6^	2.80	1.48
13	6.3%	985A>G	IVS^6^	7.23	2.51
14	7.5%	823A>G	823A>G	1.90	2.57
15	9.3%	157C>T	157C>T		1.65
16	10.2%	985A>G	199T>C	4.27	
17	11.8%	985A>G	199T>C	1.82	
18	13.9%	1140ins>G	199T>C		0.48
19^7^	24.5%	wild-type	wild-type		
20^3^	27.2%	985A>G	wild-type		
21^3^	30.8%	wild-type	IVS^6^		
22^7^	33.5%	985A>G	wild-type		
23^4^	33.6%	985A>G	wild-type		
24^7^	47.5%	wild-type	wild-type		
25^4^	53.4%	199T>C	wild-type		
26^7^	69.9%	wild-type	wild-type		
27^7^	76.1%	wild-type	wild-type		
28^7^	76.5%	wild-type	wild-type		

Case 1 to 18 were identified in NBS, with the exception of 4 and 8.

1 Relative residual MCAD enzyme activities are presented as a percentage of the mean of lymphocytes from healthy control (2.38 nmol · min^−1^ · mg^−1^, n = 6).

2 First octanoylcarnitine specimen (C8:0 I) obtained on day 2–5 of life and subsequent repeat specimen (C8:0 II) are shown in µmol · L^−1^, cut-off was set at 0.30.

3 Parents of 12.

4 Parents of 16.

5 Intervening sequences: IVS2-32C>G, IVS3+10T>C, IVS5+32C>G, IVS7-22C>A.

6 Intervening sequences: IVS2-32C>G, IVS3+10T>C, IVS5+32C>G, IVS6-14A>G and IVS7-22C>A.

7 subjects with clinical suspicion of MCADD.

### Genotypes of MCAD-deficient patients

Mutation analysis revealed that 8 out of 18 patients were 985A>G homozygotes (Table 1). Cases 4 and 8 presented with an insertion at position 245, as reported previously by Gregersen *et al.* and Maier *et al.* [14], [15], and were heterozygous for a splicing mutation (IVS9+2T>C). In addition, multiple other intronic sequence variants (IVS) were observed (IVS2-32C>G, IVS3+10T>C, IVS5+32C>G, IVS7-22C>A). Case 6, in addition to mutation 985A>G, carried the 347G>A mutation, a mutation previously described as severe [14], [16]. To the best of our knowledge, case 14 carried a novel mutation and was homozygous for 823A>G. Case 15 was a homozygous carrier of the 157C>T mutation, reported earlier [14]–[16]. Cases 16 and 17 were compound heterozygous for mutations 985A>G and 199T>C, as reported earlier [11], [14]. An insertion at position 1140 was identified in case 18 and was also previously not reported. In cases 18, 24, 26–28 a FAOD was clinically suspected but the subjects turned out not to carry a mutation on both *ACADM* alleles. Case 21, parent of case 12, had multiple heterozygous intervening sequences (IVS2-32C>G, IVS3+10T>C, IVS5+32C>G, IVS6-14A>G and IVS7-22C>A). Cases 12 and 13 originated from different families and were both heterozygous carriers of the same above described intervening sequences, in addition to their classical 985A>G mutation (Table 1).

### Correlation of genotype with MCAD residual activity and C8:0-carnitine concentration

**Figure 3:**
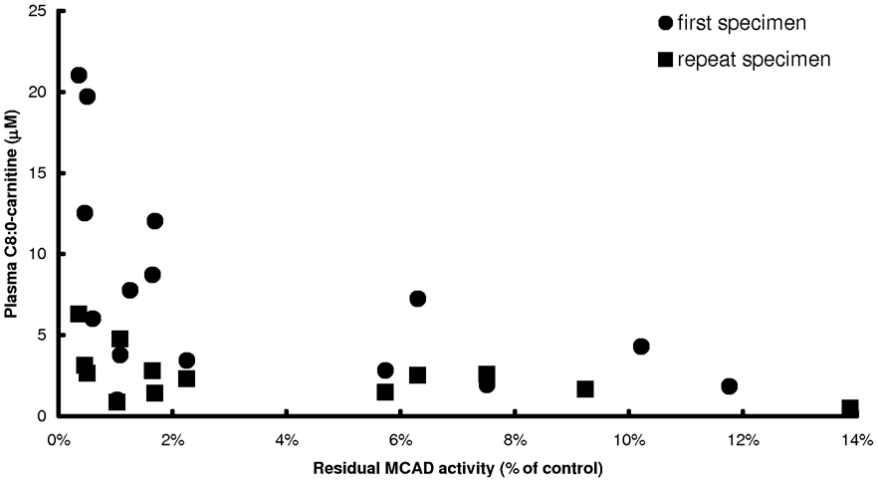
Plasma octanoyl-carnitine (C8:0) concentrations determined in dried blood spots during initial newborn screening and subsequent follow-up from patients carrying two confirmed *ACADM* mutations.

As indicated in Table 1, initial C8:0-carnitine does not always correlate with genotype and residual enzyme activity. However, a high C8:0-carnitine above >10 µM was only observed in MCADD patients with residual MCAD activities below 2% of controls and the homozygous 985A>G mutation (Fig. 3).

## Discussion

Our newly developed tandem MS method for the determination of MCAD- and VLCAD-enzyme activities in human lymphocytes is able to provide ample sensitivity to correctly and rapidly confirm diagnosis of the respective disorder in individuals identified by newborn screening. This technique is of great importance because long-chain acylcarnitines are elevated in healthy children or VLCAD heterozygotes, due to activated fatty acid oxidation in the first 2–3 days of life, and are thus interfering with NBS results. In case of MCADD, this assay also offers the possibility to predict severity of expected clinical phenotype based on residual MCAD activities and correlating genotype.

As the LC-MS/MS analytical apparatus is available in all NBS laboratories worldwide, it was important to develop an analysis method that can be used with available NBS instruments. This newly developed method thus offers the possibility to determine MCAD or VLCAD activity in one single setup, without exchanging columns, and lacking complex gradients.

### Residual VLCAD activity in VLCADD patient lymphocytes

Enzyme activities of VLCAD in human lymphocytes from healthy controls were 6.95±0.42 nmol · min^−1^ · mg protein^−1^ (n = 36) and are similar to control values reported previously by other laboratories [17]–[19]. Figure 2 clearly shows that heterozygous carriers can not be discriminated from healthy controls in all cases. However, most importantly, VLCADD patients confirmed by molecular analysis of the *ACADVL* gene can unequivocally be identified based on the determination of residual VLCAD activity in isolated lymphocytes.

### Correlation of residual MCAD activity with *ACADM* genotype

Unlike VLCADD with molecular heterogeneity, MCAD-deficiency possesses the prevalent 985A>G mutation and, since NBS, the less common 199T>C mutation. The total number of individual *ACADM* mutations reported so far is approx. 25, as reviewed in [14]. We therefore, attempted to correlate residual MCAD-activities with our *ACADM* genotyping results. This was not pursued for VLCAD activities and corresponding mutations in the *ACADVL* gene, also because of much greater molecular heterogeneity and due the limited number of observed homozygous *ACADVL* mutation carriers.

MCAD activities in healthy controls were 2.38±0.18 nmol · min^−1^ · mg protein^−1^, (n = 6) being in agreement with previous reports [17], [20], [21]. The MCAD assay was applied in lymphocyte samples from 28 subjects with suspected MCAD-deficiency. In 18 individuals, diagnosis was confirmed by mutational analysis (Table 1). All patients with the homozygous, classical 985A>G mutation had residual MCAD activities below 2.5%, therefore well in line with previous reports that identify the homozygous status of the 985A>G mutation as severe clinical phenotype [11], [16]. The 985A>G mutation was the prevalent MCAD mutation before the NBS era and patients were identified because of hypoglycaemia or hepatopathy [16]. In addition to mutation 985A>G, one MCADD patient was carrier of the 347G>A mutation, a mutation previously described as severe [14], [16], this again is in excellent agreement with a low residual MCAD activity of 1.1%. The more recent 199T>C mutation [14] was identified in a significant number of children since implementation of NBS and is considered to induce a much milder MCADD variant [14]. In our cohort, this mutation was identified three times, two in combination with the 985A>G mutation, and all three resulted in significantly higher residual MCAD activities of approx. 11%, as compared to patients being homozygous for the 985A>G mutation. Also demonstrating that MCADD severity, in case of compound heterozygosity, is determined by the mutation that is known to be the mildest of the two, in this case the 199T>C mutation [14]. In addition, patients with a heterozygous 245insT mutation and a second splicing IVS9+2T>C mutation had comparably lower residual activities of <2.5%. For the homozygous 157C>T mutation, reported earlier by [14]–[16] to result in a mild clinical phenotype, enzymatic analysis now revealed that this mutation leads to 9.3% residual MCAD activity. Interestingly, two unrelated cases presented with the 985A>G mutation plus identical mutations in intervening *ACADM* sequences (IVS2-32C>G, IVS3+10T>C, IVS5+32C>G, IVS6-14A>G and IVS7-22C>A) and both had residual MCAD activities of 6%. Heterozygous carriers of the 985A>G mutation without a second mutation (cases 20, 22 and 23) had residual MCAD activities of 24% or higher (Table 1).

### The use of plasma octanoyl-carnitine levels in newborn screening for MCADD

In our hands, measured plasma octanoyl-carnitine (C8:0) levels, based on the initial newborn screening dried blood spot, indeed functions as sensitive and specific marker for MCADD (Fig. 3). However, the C8:0-carnitine values measured are unable to discriminate between MCADD of different severity. This also applies to the repeat specimen upon confirmatory second screening, being well in line with a previous report from Chace and co-workers [22].

### Possible limitations of this study

The use of isolated peripheral blood lymphocytes may well not be fully representative for all individual organs that are differentially afflicted by MCADD or VLCADD. Of note, studies in fibroblasts would obviously present similar limitations. Furthermore, the use of ferrocenium as artificial oxidizing agent in our *in-vitro* assay, instead of electron transfer flavoprotein *in-vivo*, may possibly obscure alterations in MCAD and VLCAD activity induced by mutations that impair flavin adenine dinucleotide binding and/or electron transfer flavoprotein interaction.

### Conclusions

Taken together, we have developed a new assay that allows fast and reliable determination of MCAD and VLCAD residual activity in human lymphocytes. This method takes advantage of LC-MS/MS detection and can be implemented in any NBS laboratory using LC-MS/MS technique. Both methods provide reference values for MCAD and VLCAD enzyme activity in lymphocytes from healthy controls that correspond well with published rates in literature. Importantly, both methods provide ample sensitivity to discriminate not only patients from healthy individuals but, in case of MCADD, this method also offers the possibility to differentiate between clinical phenotypes of different severity based on residual MCAD activities, in line with their genotype.
